# Signal Transduction Proteins in *Acinetobacter baumannii*: Role in Antibiotic Resistance, Virulence, and Potential as Drug Targets

**DOI:** 10.3389/fmicb.2019.00049

**Published:** 2019-01-30

**Authors:** P. Malaka De Silva, Ayush Kumar

**Affiliations:** ^1^Department of Microbiology, University of Manitoba, Winnipeg, MB, Canada; ^2^Manitoba Chemosensory Biology Group, University of Manitoba, Winnipeg, MB, Canada

**Keywords:** two-component systems, PmrAB, AdeRS, BfmRS, stress

## Abstract

*Acinetobacter baumannii* is a notorious pathogen in health care settings around the world, primarily due to high resistance to antibiotics. *A. baumannii* also shows an impressive capability to adapt to harsh conditions in clinical settings, which contributes to its persistence in such conditions. Following their traditional role, the **T**wo **C**omponent **S**ystems (TCSs) present in *A. baumannii* play a crucial role in sensing and adapting to the changing environmental conditions. This provides *A. baumannii* with a greater chance of survival even in unfavorable conditions. Since all the TCSs characterized to date in *A. baumannii* play a role in its antibiotic resistance and virulence, understanding the underlying molecular mechanisms behind TCSs can help with a better understanding of the pathways that regulate these phenotypes. This can also guide efforts to target TCSs as novel drug targets. In this review, we discuss the roles of TCSs in *A. baumannii*, their molecular mechanisms, and most importantly, the potential of using small molecule inhibitors of TCSs as potential novel drug targets.

## Introduction

*Acinetobacter baumannii* is a Gram-negative coccobacillus, which is an important opportunistic human pathogen that causes hospital-acquired infections (Peleg et al., 2008a, 2012; Visca et al., 2011; Wong et al., 2017). Clinical importance of *A. baumannii* is emphasized by the fact that it is listed by the WHO as the “top priority” pathogen that urgently need novel and effective therapeutic options (http://www.who.int/medicines/publications/WHO-PPL-Short_Summary_25Feb-ET_NM_WHO.pdf). The success of *A. baumannii* in hospital environments can be mainly attributed to its ability to display multi-drug resistant phenotypes due to the rather robust acquisition of antibiotic resistance mechanisms (Dijkshoorn et al., 2007; Antunes et al., 2014). These include antibiotic modifying enzymes, decreased permeability to antibiotic molecules, and efflux pumps that extrude the antibiotic molecules out to the periplasm and beyond (Gordon and Wareham, 2010; Lee et al., 2017).

Multi- and pan-drug resistance in *A. baumannii* is an alarming development for healthcare facilities around the world (Rodriguez-Bano et al., 2004; Agodi et al., 2010; Sievert et al., 2013; Labarca et al., 2016). As a result, some infections caused by multi-drug resistant *A. baumannii* have become virtually untreatable with our current arsenal of antibiotics (Maragakis and Perl, 2008). Further, without any new antibiotics for Gram-negative bacteria, such as *A. baumannii* in the developmental pipeline, we are on the verge of a post-antibiotic era where even a minor infection could have lethal consequences for the patient (Xie et al., 2018).

Apart from its multidrug resistance, the success of *A. baumannii* can also be attributed to its ability to survive and persist in the harsh conditions found within hospital environmental niches (Jawad et al., 1998; Rajamohan et al., 2010). Constant and prolonged exposure to antiseptics and desiccating agents, endurance of less than optimal temperatures, and sudden changes of the environmental and nutritional conditions when transferred into the human body from an abiotic surface are some of the challenges that *A. baumannii* faces in its role as an opportunistic human pathogen. Therefore, in order to be a successful pathogen, *A. baumannii* needs to sense and adapt to these changes in an efficient and timely manner.

Signal transduction mechanisms in bacteria play a crucial role in adapting to environmental changes. TCSs are one of the most ubiquitous signal transduction systems present in bacteria that help them sense and adapt to the environmental conditions (Alm et al., 2006; Wood et al., 2018). TCSs therefore play a role in bacterial adaptive responses which can lead to the modulation of their antibiotic susceptibility and virulence. Consequently, these systems are vital to study in order to understand the mechanisms of antibiotic resistance and virulence in bacteria (Poole, 2012; Kroger et al., 2016; Schaefers et al., 2017; Kenney, 2018; Lingzhi et al., 2018). Further, TCSs can also serve as an attractive target when developing anti-virulence therapeutics (Gotoh et al., 2010b). In this review, we describe the roles of TCSs in the resistance and virulence of *A. baumannii* and their potential to be used as novel therapeutic targets.

## Two Component Systems (TCSs)

**Figure 1:**
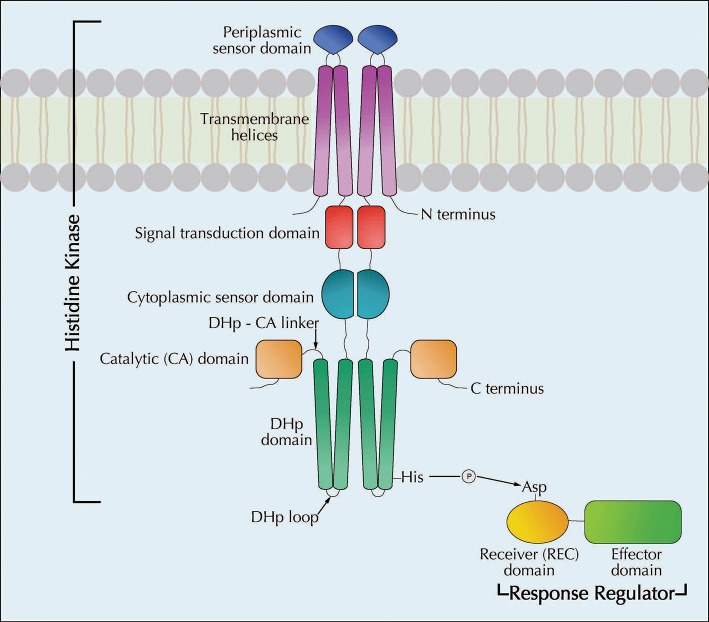
Schematic diagram showing the cellular architecture of a typical two-component regulatory system as well the mechanism of phosphotransfer between two components (modified with permission from Springer Nature Du et al., 2018. A prototypical TCS, comprised of a membrane-bound sensory **h**istidine **k**inase (HK) and a cytosolic **r**esponse **r**egulator (RR) protein, is shown. The basic mechanism of a TCS involves the HK sensing the environmental changes and relaying the message to the RR effectively through phosphorelays to initiate the necessary response. HK proteins, usually dimers, possess several conserved domains that are essential for their function, such as **d**imerization and **h**istidine **p**hosphotransfer (DHp) domain and **c**atalytic **A**TP binding (CA) domain which make up the catalytic core of the HK (Bhate et al., 2015). The H-box containing the conserved histidine residue, that gets phosphorylated, is located in the DHp domain (Casino et al., 2009, 2010). The CA domain binds ATP and phosphorylates the histidine residue, thus initiating the HK autophosphorylation (Zschiedrich et al., 2016). The DHp and CA domains are conserved among all HK proteins and the sensory domains are variable conferring specificity of signal recognition. The phosphoryl group from the H-box of the HK is ultimately transferred to a conserved aspartate residue of the receiver (REC) domain of the cognate RR thus activating the RR (Yamamoto et al., 2005). While the REC domain is highly conserved, the effector domains of RR display variability conferring specificity to the protein (Zschiedrich et al., 2016). Following its activation, dephosphorylation of the RR is critical to maintain the efficient regulatory capacity of the TCSs (Kenney, 2010). This is achieved through the phosphatase activity of the HK (Hsing and Silhavy, 1997).

TCSs are the most widespread signal transduction system present in bacteria and archaea (Stock et al., 2000). Typically, a TCS consists of two components, a histidine kinase (HK) and a response regulator (RR) (Figure 1). A high level of specificity with the HK and the RR is observed within the TCSs of a bacterial cell (Szurmant et al., 2007). However, there are instances where a single HK protein can have multiple cognate RR proteins (Lopez-Redondo et al., 2010) or when a single RR protein can be activated by multiple HK proteins (Laub and Goulian, 2007). Since their first description in 1986 (Nixon et al., 1986), an enormous amount of both HK and RR proteins have been discovered and characterized in a wide variety of bacteria (Whitworth and Cock, 2009). It is estimated that an average bacterial genome can contain up to 50–60 TCS-encoding genes (Whitworth, 2008; Whitworth and Cock, 2008; Wuichet et al., 2010). Given the advancement in bioinformatics and next generation sequencing techniques, specific databases dedicated to TCSs have become available that provide valuable information about these proteins (Ulrich and Zhulin, 2007; Barakat et al., 2011).

The TCSs in bacterial systems have implications for a wide variety of regulatory functions relating to sensing and adapting to their environment. In pathogenic bacteria, these functions often include but are not limited to antibiotic susceptibility modulation and virulence-related phenotypes, such as biofilm formation and motility (Tiwari et al., 2017).

## TCSs IN *Acinetobacter baumannii*

**Figure 2:**
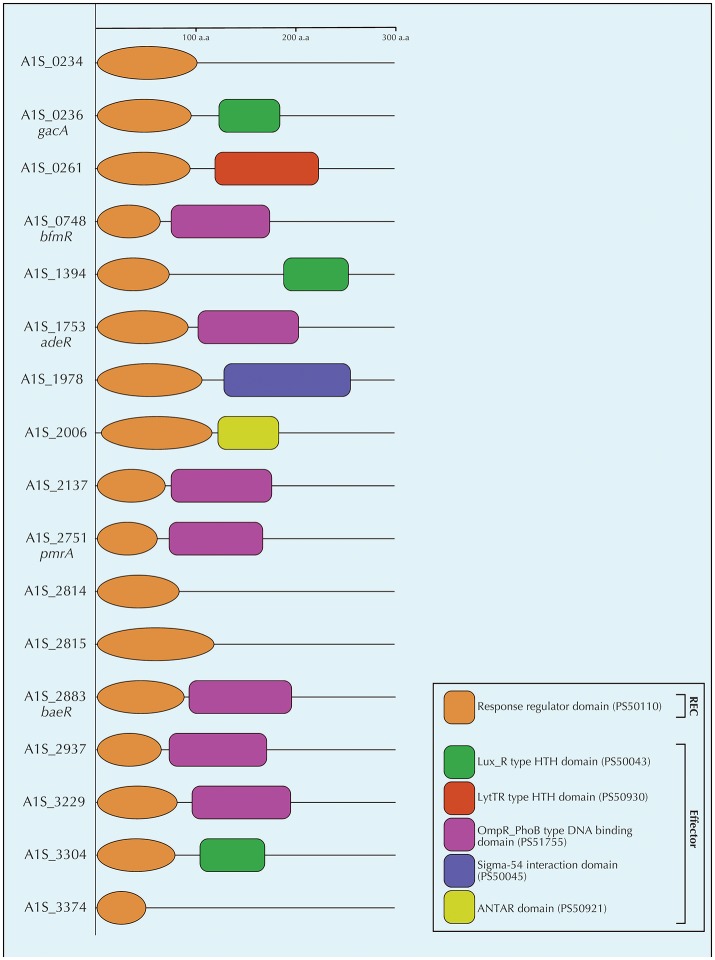
Schematic diagram of the conserved domains of all the response regulators of *A. baumannii* ATCC17978 as determined by a ScanProsite (de Castro et al., 2006). The figure depicts the receiver domain (orange) and the different effector domains identified by ScanProsite (Lux_R type HTH domain in green, LytTR type HTH domain in red, OmpR_PhoB type DNA binding domain in violet, Sigma-54 interaction domain in purple, and ANTAR domain in yellow). Most abundant effector domain was the OmpR_PhoB type DNA binding domain which was present in seven response regulators followed by the Lux_R type HTH domain which was present in three response regulators. The other three types of effector domains were exclusive to single response regulators. The numbers in parenthesis refer to the PROSITE accession numbers of the respective domains. The hybrid sensor kinase A1S_2811 was not included in the figure due to the lack of a distinct response regulator protein.

An overview of various genomes of well-characterized *A. baumannii* clinical isolates show the presence of close to 20 different genes/operons that encode for TCSs (Table 1). Most of these genes and operons have a high degree of conservation at nucleotide level, indicating that they may be involved in the important functions. However, as mentioned above, the effector domains of *A. baumannii* RR proteins can be quite diverse which is shown in Figure 2. Below we describe the TCSs in *A. baumannii* that have been characterized to date.

**Table 1:** Conservation of the TCSs in *A. baumannii* in select sequenced and publicly available clinical strains.

**Gene**	**17978mff CP012004.1**		**AB030 CP009257.1**		**Ab04-mff CP012006.1**		**LAC4 CP007712.1**		**AB0057 CP001182.2**		**AB5075-UW CP008706.1**		**AB031 CP009256.1**		**AYE CU459141.1**	
	**Identity**	**Nucleotide conservation**	**Identity**	**Nucleotide conservation**	**Identity**	**Nucleotide conservation**	**Identity**	**Nucleotide conservation**	**Identity**	**Nucleotide conservation**	**Identity**	**Nucleotide conservation**	**Identity**	**Nucleotide conservation**	**Identity**	**Nucleotide conservation**
*A1S_0234*	99%	446/447	98%	438/447	97%	434/447	97%	434/447	98%	437/447	98%	437/447	93%	417/447	98%	437/447
*A1S_0235*	100%	1,524/1,524	98%	1,497/1,524	99%	1,505/1,524	99%	1,506/1,524	98%	1,501/1,524	98%	1,501/1,524	98%	1,500/1,524	98%	1,501/1,524
*A1S_0236 gacA*	100%	636/636	99%	634/636	99%	633/636	99%	632/636	99%	631/636	99%	631/636	99%	631/636	99%	631/636
*A1S_0260*	100%	1,140/1,140	99%	1,134/1,140	99%	1,132/1,140	99%	1,132/1,140	99%	1,128/1,140	99%	1,128/1,140	99%	1,131/1,140	99%	1,128/1,140
*A1S_0261*	100%	741/741	99%	737/741	99%	736/741	99%	735/741	99%	733/741	99%	733/741	99%	736/741	99%	733/741
*A1S_0574 gacS*	100%	2,808/2,808	99%	2,780/2,808	99%	2,769/2,808	99%	2,769/2,808	99%	2,773/2,808	99%	2,773/2,808	99%	2,773/2,808	99%	2,773/2,808
*A1S_0748 bfmR*	100%	717/717	99%	713/717	99%	708/717	99%	708/717	99%	713/717	99%	713/717	100%	717/717	99%	713/717
*A1S_0749 bfmS*	100%	1,593/1,593	98%	1,564/1,593	97%	1,550/1,593	97%	1,550/1,593	98%	1,566/1,593	98%	1,566/1,593	99%	1,587/1,593	98%	1,566/1,593
*A1S_1393*	100%	3,465/3,465	94%	3,261/3,465	94%	3,258/3,465	94%	3,257/3,465	95%	3,299/3,465	95%	3,299/3,465	95%	3,303/3,465	95%	3,298/3,465
*A1S_1394*	100%	960/960	96%	920/960	99%	952/960	99%	951/960	97%	928/960	97%	928/960	96%	922/960	97%	928/960
*A1S_1753 adeR*	100%	744/744	98%	731/744	–	–	–	–	99%	735/744	99%	735/744	97%	721/744	99%	735/744
*A1S_1754 adeS*	100%	1,086/1,086	98%	1,041/1,065	–	–	–	–	97%	1,052/1,086	97%	1,052/1,086	Disrupted	Disrupted	97%	1,052/1,086
*A1S_1977*	100%	1,110/1,110	99%	1,094/1,110	99%	1,094/1,110	99%	1,094/1,110	98%	1,093/1,110	98%	1,093/1,110	99%	1,096/1,110	98%	1,093/1,110
*A1S_1978*	100%	1,494/1,494	97%	1,454/1,494	97%	1,452/1,494	97%	1,451/1,494	97%	1,448/1,494	97%	1,447/1,494	97%	1,449/1,494	97%	1,448/1,494
*A1S_2006*	100%	591/591	Disrupted		100%	591/591	100%	591/591	99%	588/591	99%	588/591	99%	588/591	99%	588/591
*A1S_2137*	100%	717/717	93%	669/717	95%	674/711	95%	673/711	95%	681/717	95%	681/717	98%	703/717	95%	680/717
*A1S_2138*	100%	2,655/2,655	95%	2,526/2,656	94%	2,509/2,656	95%	2,510/2,656	96%	2,537/2,656	96%	2,537/2,656	94%	2,502/2,656	96%	2,538/2,656
*A1S_2287*	99%	1,358/1,360	99%	1,342/1,360	99%	1,356/1,360	99%	1,355/1,360	98%	1,336/1,360	98%	1,336/1,360	99%	1,344/1,360	98%	1,336/1,360
*A1S_2750 pmrB*	100%	1,335/1,335	99%	1,318/1,335	99%	1,330/1,335	99%	1,330/1,335	99%	1,316/1,335	99%	1,316/1,335	99%	1,318/1,335	99%	1,316/1,335
*A1S_2751 pmrA*	100%	675/675	99%	670/675	99%	665/675	99%	665/675	99%	668/675	99%	668/675	99%	671/675	99%	668/675
*A1S_2811*	100%	4,521/4,521	98%	4,426/4,521	98%	4,441/4,521	98%	4,442/4,521	98%	4,433/4,521	98%	4,433/4,521	98%	4,442/4,521	98%	4,433/4,521
*A1S_2814*	100%	363/363	99%	361/363	99%	362/363	99%	362/363	99%	362/363	99%	362/363	99%	362/363	99%	362/363
*A1S_2815*	100%	384/384	99%	383/384	99%	381/384	99%	381/384	99%	383/384	99%	383/384	99%	383/384	99%	383/384
*A1S_2883 baeR*	100%	687/687	99%	680/687	99%	684/687	99%	684/687	99%	678/687	99%	678/687	99%	679/687	99%	678/687
*A1S_2884 baeS*	99%	1,463/1,464	99%	1,458/1,464	99%	1,457/1,464	99%	1,456/1,464	99%	1,454/1,464	99%	1,454/1,464	99%	1,449/1,464	99%	1,454/1,464
*A1S_2906*	100%	1,269/1,269	99%	1,250/1,269	99%	1,256/1,269	99%	1,256/1,269	99%	1,255/1,269	99%	1,255/1,269	99%	1,255/1,269	99%	1,255/1,269
*A1S_2937*	100%	684/684	–	–	85%	579/684	100%	684/684	87%	592/684	87%	592/684	–	–	87%	592/684
*A1S_2938*	100%	1,374/1,374	–	–	–	–	99%	1,372/1,374	91%	1,256/1,377	91%	1,256/1,377	–	–	91%	1,256/1,377
*A1S_3229*	100%	765/765	99%	760/765	99%	757/765	99%	757/765	99%	756/765	99%	756/765	99%	761/765	99%	756/765
*A1S_3230*	100%	1,458/1,458	99%	1,444/1,458	99%	1,445/1,458	99%	1,445/1,458	99%	1,440/1,458	99%	1,440/1,458	99%	1,452/1,458	99%	1,440/1,458
*A1S_3302*	100%	3,498/3,498	98%	3,441/3,498	99%	3,450/3,498	99%	3,450/3,498	98%	3,434/3,498	98%	3,434/3,498	98%	3,432/3,498	98%	3,434/3,498
*A1S_3304*	100%	651/651	99%	644/651	99%	645/651	99%	645/651	99%	643/651	99%	643/651	99%	645/651	99%	643/651
*A1S_3374*	100%	164/164	100%	164/164	99%	163/164	99%	163/164	100%	164/164	100%	164/164	100%	164/164	100%	164/164
*A1S_3376*	100%	1,359/1,359	99%	1,339/1,359	98%	1,329/1,359	98%	1,329/1,359	98%	1,331/1,359	98%	1,331/1,359	99%	1,341/1,359	98%	1,331/1,359

*The list of putative TCSs were extracted from the P2CS database (www.p2cs.org) from the A. baumannii ATCC17978 strain (Accession number: CP000521.1)*.

### AdeRS

**Figure 3:**
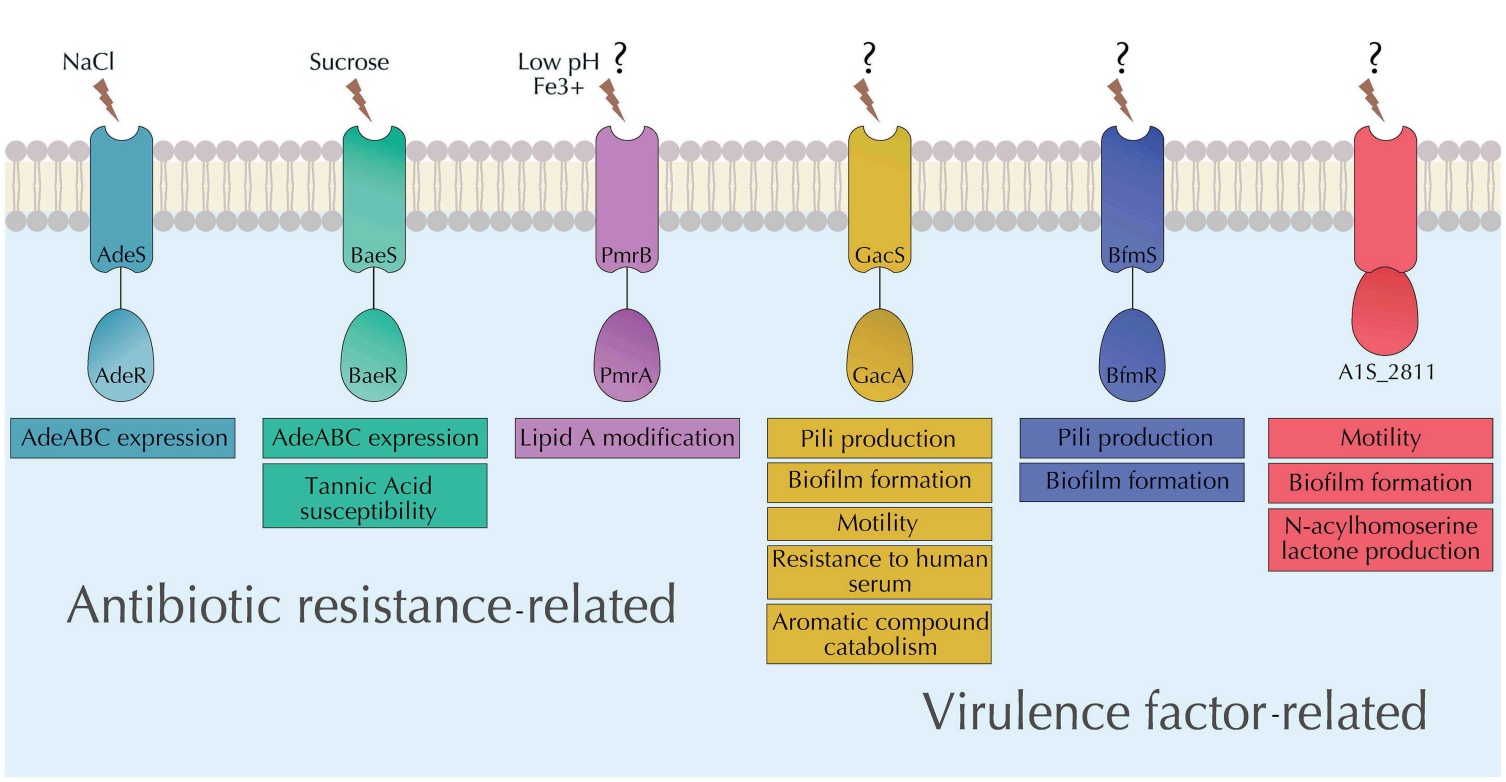
Summary of the characterized TCSs in *A. baumannii*. Functions of each of the characterized TCSs in *A. baumannii* (AdeRS, BaeSR, PmrAB, GacSA, BfmRS, and A1S_2811) as well as their known stimuli are depicted.

AdeRS is the first characterized and also the most studied TCS in *A. baumannii*. It was first described in a clinical strain *A. baumannii* BM4454, when the inactivation of *adeS* resulted in an increased susceptibility to aminoglycosides due to the downregulation of the RND efflux pump AdeABC (Marchand et al., 2004) (Figure 3). Since it was first identified, a number of mutations in either *adeR, adeS*, or both have been shown to be directly responsible for the overexpression of the AdeABC pump (Ruzin et al., 2007; Yoon et al., 2013; Sun et al., 2016). Considering AdeRS system's role in the expression of AdeABC, it can be said that it plays a role in the susceptibility of *A. baumannii* to antibiotics that are substrates of the AdeABC pump. Further, the overexpression of AdeABC efflux pump has been associated with the decreased susceptibility to tigecycline observed in some clinical isolates of *A. baumannii* (Sun et al., 2014; Yuhan et al., 2016) thus implicating an indirect role of AdeRS in the susceptibility toward tigecycline. This is important since tigecycline is one of the last resort antibiotics for the treatment of multidrug resistant *A. baumannii* infections (Ni et al., 2016). However, there needs to be further investigations into this due to the possibility of involvement of other factors for the observed tigecycline susceptibility (Yoon et al., 2013).

Recent transcriptomics data suggest that the role of AdeRS extends well-beyond the expression of AdeABC efflux pump. A study in *A. baumannii* AYE showed that AdeRS controls the expression of almost 600 different genes (Richmond et al., 2016). Products of a number of these genes are believed to play a role in virulence, biofilm formation and multi drug efflux activity. However, deletion of *adeB* in the same strain resulted in similar phenotypes as deletion of *adeRS*. This suggests that at least some phenotypic changes observed upon the *adeRS* deletion may be a result of the decreased expression of the AdeABC efflux pump (Richmond et al., 2016).

The multifaceted regulon of AdeRS remains to be explored further, especially in clinically relevant phenotypes of *A. baumannii*. Further, environmental signals that activate the sensor kinase, AdeS, remain mostly unknown. However, we recently uncovered evidence that AdeRS system maybe responding to the NaCl concentrations in the growth medium (De Silva and Kumar, 2017). This work links adaption to environmental conditions, such as NaCl concentration to antibiotic susceptibility (as a result of expression of the AdeABC pump) as well as virulence factors, such as biofilm formation and surface-associated motility. It is therefore obvious that AdeRS plays a role in the antibiotic susceptibility of *A. baumannii* but also possibly in its virulence. However, it's role in antibiotic susceptibility and virulence is likely to be more strain-specific, as it is not uncommon to find disrupted copies of *adeRS* genes in clinical isolates of *A. baumannii*, such as LAC-4 and AB031 (Table 1).

### BaeSR

BaeSR, named such because of its homology with an *E. coli* TCS (Leblanc et al., 2011), mediates a possible “cross–talk” with other TCSs. It has been shown to regulate overlapping regulons with other TCSs in *A. baumannii*. BaeSR was initially thought to be associated with the regulation of AdeABC RND efflux pump expression (Lin et al., 2014) (Figure 3). This is indicative of a possible cross–talk between BaeSR and AdeRS. Further investigations into the BaeSR revealed that it may also modulate the expression of AdeIJK and MacAB-TolC efflux pumps (Henry et al., 2012). However, efforts to determine the DNA binding sites in the promoters corresponding to the observed target genes remain unsuccessful, leaving room for further explorations (Lin et al., 2015). A phenotypic microarray screen revealed that the deletion of *baeR* resulted in reduced tolerance of *A. baumannii* to tannic acid (Lin et al., 2015), a diverse group of natural antibacterial compound (Henis et al., 1964). Tannic acids has been used as a topical agent in burn patients (Hupkens et al., 1995) as they display effective antibacterial activity against various bacteria, including *E. coli, Staphylococcus aureus, Staphylococcus epidermidis, Salmonella* spp. etc (Kim et al., 2016). Tannic acid has also been shown to inhibit biofilm formation in *Staphylococcus aureus* (Payne et al., 2013). In *A. baumannii*, Tannic acids are being explored as adjuvants for antimicrobial therapy. They were shown to synergize the activity of novobiocin, rifampicin, and fusidic acid against MDR *A. baumannii* (Chusri et al., 2009). However, the role of BaeSR TCS in tannic acid as well as the expression of efflux pumps controlled by BaeSR will have to be considered to in order to explore the clinical usage of tannic acid as an adjuvant therapy options against *A. baumannii*.

Studies on the environmental signals that BaeSR responds to remain limited. However, expression of *baeR* and *baeS* in *A. baumannii* is induced by sucrose (20% w/v) (Lin et al., 2014), suggesting that BaeSR may be involved in *A. baumannii*'s response to osmotic stress.

### PmrAB

*A. baumannii*'s resistance to commonly used antibiotics has led to an increased use of “last resort” antibiotics, such as colistin (Karaiskos et al., 2017; Jiménez-Guerra et al., 2018). As a result, emergence of colistin resistance is becoming more common in *A. baumannii* (Cai et al., 2012; Lean et al., 2015). Investigations into the mechanisms of resistance to colistin in *A. baumannii* have revealed the involvement of PmrAB resistance (Park et al., 2011; Rolain et al., 2013), named so for its role in polymixin (Figure 3). PmrAB has been described in various Gram-negative pathogens including *E. coli* (Quesada et al., 2015), *Salmonella enterica* (Gunn, 2008)*, Klebsiella pneumoniae* (Cheng et al., 2010), and *Pseudomonas aeruginosa* (Lee and Ko, 2014) and has been shown to have a similar function colistin resistance. Observations of mutations in both *pmrA* (RR) and *pmrB* (HK) leading to decreased susceptibility to colistin presented preliminary evidence of the connection between PmrAB and colistin susceptibility in *A. baumannii* (Adams et al., 2009). Further, both colistin-resistant clinical isolates as well as laboratory generated spontaneous mutants showed phosphoethanolamine modification of lipid A of lipopolysaccharide (LPS) within the outer membrane (Arroyo et al., 2011; Beceiro et al., 2011). The modification of lipid A is mediated by PmrC which is generally part of the same operon as *pmrAB* (Raetz et al., 2007). PmrC can add phosphoethanolamine to either 4′ or 1′ phosphate of lipid A (Da Silva and Domingues, 2017). This modification of LPS results in a positively charged phosphate groups and prevents the binding of the cationic colistin (Tamayo et al., 2005a,b; Arroyo et al., 2011). Mutations in both *pmrA* and *pmrB* cause the overexpression of the *pmrCAB* operon.

Observations that low pH or supplementation of Fe^3+^ in the growth medium (Adams et al., 2009) lead to colistin resistance may suggest that PmrB could be responding to those signals (Gunn, 2008). However, growth of *A. baumannii* under low pH or in iron supplemented growth media failed to alter the expression of *pmrA* (Adams et al., 2009). Therefore, the environmental signals to which PmrAB responds to in *A. baumannii* remain elusive.

### GacSA

GacSA is a TCS that is well-characterized in *Pseudomonas sp*. (Gooderham and Hancock, 2009). GacSA in *A. baumannii* ATCC19606 was identified when the transposon insertions in the *gacS* sensor kinase gene rendered the mutants incapable of utilizing citrate as the sole carbon source (Dorsey et al., 2002). This suggests that GacSA is involved in citrate metabolism. Since the initial characterization of GacSA in *A. baumannii* ATCC19606, a number of subsequent studies have carried out the functional characterization of GacSA TCS in *A. baumannii* ATCC17978. Interestingly, in *A. baumannii* ATCC17978, the *gacS* gene is not linked to the response regulator-encoding gene. Rather, it has both a HisKA domain and a REC domain suggesting that it could function as a hybrid sensor kinase. Although, there is also a possibility that in *A. baumannii* ATCC17978, the response regulator for GacS is encoded elsewhere in the genome. This indicates that the organization of the *gacSA* genes may vary from strain to strain in *A. baumannii*.

In addition to the initially observed metabolic role of *gacS*, a transposon mutant with a disrupted *gacS* gene displayed significantly reduced *A. baumannii*'s ability to inhibit *Candida albicans* (Peleg et al., 2008b). *gacS* deletion mutant also displayed attenuated virulence in a mouse infection model (Cerqueira et al., 2014). Deletion of *gacS* also led to the revelation of its involvement in a number of other virulence related functions. These include control of pili synthesis, motility, and biofilm formation, resistance against human serum, and metabolism of aromatic compounds (Cerqueira et al., 2014) (Figure 3). GacSA is involved in the regulation of the aromatic compound catabolism through the *paa* operon that encodes the components of the phenylacetic acid catabolic pathway. The *paa* gene cluster is significantly downregulated in the *gacSA* deletion mutants which may explain their attenuated virulence in a mouse septicaemia model (Cerqueira et al., 2014). The attenuated virulence of *gacSA* deletion mutants was observed in a later study involving a zebra fish virulence model as well (Bhuiyan et al., 2016) adding to the repertoire of studies that suggest that GacSA may function as a global virulence regulator in *A. baumannii*.

### BfmRS

Biofilm formation is an important virulence factor of pathogens, such as *A. baumannii* that helps them survive harsh conditions present in hospital environments. The ability of *A. baumannii* to form biofilms starts with its attachment to surfaces that is mediated by the expression of pili. The expression of pili is mediated by the *csu* operon in *A. baumannii* and is under the regulatory control of BfmRS (Tomaras et al., 2008). Deletion of the response regulator *bfmR* in *A. baumannii* ATCC19606 resulted in the complete abolishment of biofilm formation (Tomaras et al., 2008) (Figure 3). While of the role of *csu* operon in the attachment of *A. baumannii* on abiotic surfaces is well-established (Tomaras et al., 2003; Moon et al., 2017; Pakharukova et al., 2018), its role in the adherence of *A. baumannii* to human epithelial cells remains ambiguous. It was observed that *A. baumannii* ATCC19606 strain lacking *csuE* in fact adhered to bronchial epithelial cells better than the wild-type parent making the role pili in adherence to epithelial cells unclear (de Breij et al., 2009). It is possible that this was a strain specific outcome and further investigations are required to draw definitive conclusions.

In addition to regulating biofilm formation, BfmRS also plays a role in regulating the exopolysaccharide production (Geisinger et al., 2018). Exopolysaccharides play an important role in virulence of *A. baumannii* as they are a component of the capsule, which protects *A. baumannii* against serum killing and increasing the virulence in animal models. Further, antibiotic exposure leads to an increase in capsule production in *A. baumannii* mediated by increased expression of genes in K-locus, which in turn is regulated by the BfmRS system (Geisinger and Isberg, 2015).

Crystal structure of BfmR shows that it binds to its own promoter with higher affinity in an inactive (dephosphorylated) state compared to the active (phosphorylated) state (Draughn et al., 2018). This is unusual behavior highlights a unique self-regulation strategy of BfmRS system Therefore, BfmRS system is an excellent candidate to study not only the mechanisms that regulate virulence factors in *A. baumannii* but also the functioning of the TCSs systems in general.

### A1S_2811

A1S_2811 is a recently characterized hybrid sensor kinase possessing four histidine–containing phosphotransfer domains as well as a regulatory CheA-like domain and a CheY-like receiver domain (Chen et al., 2017). CheA and CheY homologs in *E. coli* and *P. aeruginosa* are associated with regulatory roles in controlling motility via regulating either pili or flagella (Li et al., 1995; Alon et al., 1998; Bertrand et al., 2010). Interestingly, in *A. baumannii*, this hybrid sensor kinase is expressed in an operon composed of five genes where the four other genes upstream are *pilJ, pilI, pilH*, and *pilG*. Phenotypic analysis of the deletion mutant of *A1S_2811* revealed a significant reduction in surface motility and biofilm formation at the gas-liquid interface. More intriguingly, *abaI*, which encodes a N-acylhomoserine lactone involved in quorum sensing, was also significantly downregulated. Supplementation with synthetic homoserine lactone complemented the biofilm and motility phenotypes (Figure 3). This suggests that A1S_2811 regulates biofilm formation and surface motility through an AbaI-associated quorum sensing pathway rather than the conventional pili associated pathway (Chen et al., 2017). This is in contrast to the BfmRS mediated regulon controlling biofilm formation in *A. baumannii*. Association of both BfmRS and A1S_2811 with biofilm formation is also an example of one phenotype being under the control of multiple regulatory networks formed by different TCSs.

## TCSs as Potential Novel Drug Targets in Bacterial Pathogens

Given the important role that TCSs play in regulating the clinically-relevant phenotypes (virulence and/or antibiotic resistance) of bacterial pathogens, it has been proposed that targeting them therapeutically can offer an alternate treatment strategy against multidrug resistant pathogens (Barrett and Hoch, 1998; Stephenson and Hoch, 2002a,b, 2004; Gotoh et al., 2010b; Cardona et al., 2018). TCSs in *A. baumannii* as well as other organisms offer promise as novel drug targets because of a number of reasons; (i) their conserved nature among bacteria, (ii) their involvement in modulating antibiotic resistance and virulence phenotypes, (iii) their absence in mammalian cells thus reducing off–target toxicity, (iv) lesser potential of resistance development, as the focus of the approach is to supress virulence and/or antibiotic susceptibility rather than killing the cells. It is therefore not all that surprising that TCSs from different organisms have been studied as potential therapeutic targets. Table 2 summarizes a few examples of the use of TCSs inhibitors used in bacterial pathogens other than *A. baumannii*.

**Table 2:** A brief summary of the examples of using TCS inhibitors as a therapeutic option in bacterial pathogens other than *A. baumannii*.

**TCS**	**Organism(s)**	**Inhibitor**	**Inhibitory action**	**References**
AlgR1/AlgR2	*Pseudomonas aeruginosa*	Thiazole derivatives	Inhibition of AlgR1 phosphorylation and AlgR2 kinase activity	Roychoudhury et al., 1993
WalKR	*Staphylococcus aureus Bacillus subtilis*	Walkmycin B Waldiomycin	Inhibition of autophosphorylation of WalK	Okada et al., 2010; Igarashi et al., 2013
		Walrycin A Walrycin B	Inhibition of phosphotransfer from WalR	Gotoh et al., 2010a
QseCB	Enterohemorrhagic *E. coli* (EHEC)	LED209	Inhibition of autophosphorylation of QseC	Rasko et al., 2008
PhoPQ	*Salmonella sp*.	Radicicol	Activity against PhoQ	Guarnieri et al., 2008
VanSR	*Enterococcus faecium*	Thiazole derivatives	Inhibition of phosphotransfer to VanR	Ulijasz and Weisblum, 1999

## Potential Of TCSs as Novel Drug Targets in *A. baumannii*

In *A. baumannii*, small molecule inhibitors, such as 2-aminoimidazole compounds have shown great promise in inhibiting the action of both PmrA and BfmR. The 2-aminoimidazole-based adjuvants used in combination with colistin were able to reverse colistin resistance in *A. baumannii* clinical isolates through inhibiting PmrAB and thereby abolishing the lipid A modification (Brackett et al., 2016). A promising feature of this strategy was that no resistance toward the PmrAB inhibitor was observed during the testing period of 7-days (Harris et al., 2014). Yet another example is the use of small molecule 2-aminoimidazole derivatives to inhibit the functions of BfmR (Thompson et al., 2012), such as biofilm formation (Milton et al., 2018). However, as with any other small molecule inhibitor, the cytotoxicity of the compounds used against PmrAB and BfmRS remains to be determined before the inhibitors could be deployed in a clinical setting.

Preliminary findings on the inhibition of BfmRS and PmrAB system are encouraging. In addition, AdeRS, A1S_2811, or GacSA can potentially be explored as therapeutic targets because of the important role they have been shown to play in the antibiotic resistance and virulence of *A. baumannii*.

## Challenges in Targeting TCSs For Theraputics

Despite the fact that the investigations of the TCSs show an increasing amount of information being uncovered during the recent years, the majority of these efforts have focused on the cellular functions carried out by TCSs. This has left a void of information about the environmental signals that act as a trigger for the histidine kinase stimulation. The proposed stimuli for the already characterized TCSs are limited to osmotic stress for BaeSR (Lin et al., 2014), monovalent cations for AdeRS (De Silva and Kumar, 2017); and possibly low pH and Fe^3+^ for PmrAB (Gunn, 2008; Adams et al., 2009). Uncovering the environmental stimuli that activate a TCS response is critical in understanding the molecular pathways that are used for gene regulation by a particular TCS. These pathways can then be better exploited to render *A. baumannii* non-virulent and/or antibiotic susceptible. However, it is often difficult to determine these signals due to an array of practical reasons including, but not limited to, the potential ability of sensor kinases to detect multiple stimuli and difficulty in expressing, purifying, and experimenting with histidine kinase proteins *in vitro* in their natural conformations.

## Conclusions and Future Perspectives

It is evident that the characterized TCSs present in *A. baumannii* are responsible for controlling a number of antibiotic resistance and virulence associated phenotypes, which contribute to the success of this organism as a human pathogen. Research on TCSs in *A. baumannii* has extended our knowledge on virulence and resistance mechanisms in this organism over the last few years. However, there is still a considerable knowledge gap in comprehensive understanding of the complete TCS regulatory networks. Nonetheless, TCSs present themselves as potential targets for drug design and the use of 2-aminoimidazole compounds is are encouraging. A better characterization of these systems both genetically and functionally is key for the potential use of TCS as therapeutic targets.

## Author contributions

All authors listed have made a substantial, direct and intellectual contribution to the work, and approved it for publication.

### Conflict of Interest Statement

The authors declare that the research was conducted in the absence of any commercial or financial relationships that could be construed as a potential conflict of interest.
